# Connecting two worlds: positive correlation between physicochemical approach with blood gases and pH in pediatric ICU setting

**DOI:** 10.1186/s13104-019-4770-6

**Published:** 2019-11-09

**Authors:** Chanapai Chaiyakulsil, Papope Mueanpaopong, Rojjanee Lertbunrian, Somchai Chutipongtanate

**Affiliations:** 1 Division of Pediatric Critical Care, Department of Pediatrics, Faculty of Medicine, Thammasat University, Bangkok, Thailand; 2 Department of Pediatrics, Faculty of Medicine, Ramathibodi Hospital, Mahidol University, Bangkok, Thailand; 3 Division of Pediatric Critical Care, Department of Pediatrics, Faculty of Medicine Ramathibodi Hospital, Mahidol University, Bangkok, Thailand; 4 Pediatric Translational Research Unit, Department of Pediatrics, Faculty of Medicine Ramathibodi Hospital, Mahidol University, Bangkok, Thailand; 5 Section for Clinical Epidemiology and Biostatistics, Faculty of Medicine Ramathibodi Hospital, Mahidol University, Bangkok, Thailand

**Keywords:** Acidosis, Alkalosis, Blood gas, Pediatrics, Strong ion difference

## Abstract

**Objective:**

Physicochemical approach such as strong ion difference provides a novel concept in understanding and managing acid–base disturbance in patients. However, its application in pediatrics is limited. This study aimed to evaluate a correlation between the physicochemical approach and blood gas pH for acid–base determination in critically ill pediatric patients.

**Results:**

A total of 130 pediatric patients were included, corresponding to 1338 paired measures for analyses. Of these, the metabolic subgroup (743 paired measures) was defined. Among physicochemical parameters, the effective strong ion difference showed the best correlation with the blood gas pH in the whole cohort (R = 0.398; p < 0.001) and the metabolic subgroup (R = 0.685; p < 0.001). Other physicochemical parameters (i.e., the simplified and the apparent strong ion difference, the strong ion gap, and the sodium chloride gap) and the traditional measures (standard base excess, lactate, chloride and bicarbonate) also showed varying degrees of correlation. This study revealed the positive correlation between physicochemical parameters and the blood gas pH, serving as a connecting dot for further investigations using physicochemical approach to evaluate acid–base disturbance in pediatric population.

## Introduction

Acid–base disturbances, more specifically metabolic acidosis, is one of most concerning issues found in most, if not all, critically ill patients which affect morbidity and mortality. Our understanding of acid–base physiology is continually evolving. From Latin origin of the word ‘*acidus*’ (sour taste) to Brønsted and Lowry’s definition of acid as substances that can donate a proton (H^+^) in 1923 and Henderson–Hasselbalch formula of the relationship between serum bicarbonate, pCO_2_, and pH in 1916. New understanding provides scientists and clinicians insight into the acid–base as tools to understand and treat patient’s acid–base disturbance [1].

The traditional approach of acid–base disturbances by Henderson–Hasselbalch has specific weaknesses that need to be addressed. First, there are non-bicarbonate buffers such as hemoglobin and albumin, which frequently alters in the intensive care setting. Thus, a change in bicarbonate alone might not truly reflect the total amount of non-respiratory acids and bases [2, 3]. Another traditional approach involves a concept of buffer base which takes into account of all plasma buffer anions and non-volatile, weak acid buffers (albumin and phosphate) as well as consideration of hemoglobin as a buffer [4, 5]. By incorporating these buffers, it yields the concept of base excess (BE) which represents the amount of alkali or acid that need to be added to 1 L of oxygenated blood at pCO_2_ of 40 mmHg to obtain pH of 7.4 [5]. BE might suffer inaccuracy due to pCO_2_ changes across extracellular fluid space. Thus the term of standard base excess (SBE) was introduced after standardizing the effect of hemoglobin on CO_2_ titration. Moreover, BE and SBE equation assumes normal non-buffer ions such that of albumin and phosphate. The decrease in these buffers might result in unstable BE and SBE [6].

A more recent physicochemical approach to acid–base status, introduced by Peter Stewart in 1978, challenge clinician’s understanding of acid–base physiology of human body fluid [7]. With the principle of electroneutrality and the conservation of mass, Stewart proposed that the acid–base status of body fluid is determined by three independent variables, pCO_2_, concentration of total weak acid (A_TOT_), and strong ion difference (SID), rather than serum bicarbonates, which are dependent variables [7, 8]. Since then, the physicochemical approach had been applied to understanding and treatment of various acid–base disorders. Several studies were conducted in attempt to compare the physicochemical approach with the traditional approaches in term of diagnostic abilities for classification of acid–base disorders and in the determination of prognosis in critically ill patients [2, 9–13]. Few studies were done using both traditional and physicochemical approach in the determination of acid–base disturbances in critically ill pediatric patients [1, 14, 15]. Most of this evidence, nonetheless, were based on data from adult population and the application of the physicochemical approach in pediatric patients is scant.

This study aimed to provide direct evidence of the correlation between several physicochemical approaches with blood pH in order to illustrate its importance in acid–base disorders determination in critically ill pediatric patients. The base excess model and the traditional approach of acid–base determinations were also evaluated.

## Main text

### Methods

#### Study design

This retrospective, observational study collected clinical data and laboratory results from Electronic Medical Records of pediatric patients with the age of 1 month to 15 years who were admitted to pediatric intensive care unit (PICU) at Ramathibodi Hospital during 2014–2016. Patient characteristics including age, gender, the cause of PICU admission as well as the laboratory results were reviewed. The laboratory data of a simultaneous collection of blood gas analysis (e.g., pH, pCO_2_, pO_2_) and blood chemistry (i.e., [Na^+^], [K^+^], [Cl^−^], [HCO_3_^−^], [Ca^2+^], [Mg^2+^], [PO_4_^−^], arterial lactate, and albumin), so-called the paired specimen, were collected. The Ethic Committee of Ramathibodi Hospital, Mahidol University approved this study, and informed consent was waived due to the retrospective nature of the study (protocol ID 10-57-07).

Correlation analysis was performed on the basis of the whole cohort (using all collected samples from the paired specimens) and the subgroup of metabolic acidosis or alkalosis. To exclude data with primary respiratory acid–base disturbance, in which the pH change is directly affected by carbon dioxide but not the organic/inorganic ions, any sample that show the acidic pH (< 7.4) with high HCO_3_^−^ (> 24 mmol/L) and the basic pH (> 7.4) with low HCO_3_^−^ (< 24 mmol/L) were excluded. The remaining samples were then analyzed as the metabolic subgroup.

#### Evaluation of acid–base parameters

The simplified SID and the apparent SID (SIDa) are depended on the difference between the measured strong cations and anions which represent the unmeasured anions and thus directly correlates with [H^+^] as dictated by the law of electroneutrality. The effective SID (SIDe) observes the relationship among the measured pH, bicarbonate, albumin, and phosphate, as well as the effect of remaining anions, to estimate the unmeasured cations. Increase in the simplified SID, SIDa, and SIDe would correlates with alkalosis and decrease in these parameters would signifies acidosis in patients. These physicochemical parameters were calculated as follows [2, 16, 17];

$$ {\text{SID}} = \left[ {{\text{Na}}^{ + } } \right] + \left[ {{\text{K}}^{ + } } \right]{-}\left[ {{\text{Cl}}^{ - } } \right] $$

$$ {\text{SIDa}} = \left\{ {\left[ {{\text{Na}}^{ + } } \right] + \left[ {{\text{K}}^{ + } } \right] + \left[ {{\text{Ca}}^{ + + } } \right] + \left[ {{\text{Mg}}^{ + + } } \right]} \right\}{-}\left\{ {\left[ {{\text{Cl}}^{ - } } \right] + \left[ {{\textsc{l}}{\text{-lactate}}^{ - } } \right]} \right\} $$

$$ {\text{SIDe}} = \left( {0.0 30 1*{\text{pCO}}_{ 2} * 10^{{{\text{pH-6}}. 1}} } \right) + \left\{ {\left( {\left[ {\text{albumin}} \right]*\left( {{\text{pH}}*0. 1 2 3- 0. 6 3 1} \right)} \right)} \right\} + \left\{ {\left( {\left[ {\text{phosphate}} \right]*\left( {{\text{pH}}*0. 30 9- 0. 4 6 9} \right)} \right)} \right\} $$

The strong ion gap (SIG) is the difference between the unmeasured anions and cations which were estimated from SIDa and SIDe, respectively. Increase in SIG suggests the presence of unmeasured anions. SIG was determined by the following equation;

$$ {\text{SIG}} = {\text{SIDa}}{-}{\text{SIDe}} $$

The sodium chloride (Na–Cl) gap can be considered as the SID surrogate and was calculated by the following equation;

$$ {\text{Na}}{-}{\text{Cl gap}} = \left[ {{\text{Na}}^{ + } } \right]{-}\left[ {{\text{Cl}}^{ - } } \right] $$

Standard base excess (SBE), arterial lactate, serum bicarbonate and serum chloride were included to serve as a traditional approach for evaluation of acid–base disturbances. SBE was calculated from the measured pH and HCO_3_^−^ as following;

$$ {\text{SBE}} = 0. 9 2 8 7*\left\{ {\left[ {{\text{HCO}}_{ 3}^{ - } } \right] - 2 4. 4+ 1 4. 8 3*\left( {{\text{pH-7}}. 4} \right)} \right\} $$

#### Statistical analysis

Sample size was calculated using G*Power program (http://www.gpower.hhu.de) with alpha = 0.05, power = 0.95, and the effect size of 0.1021 obtained from the preliminary data of the measured pH and the simplified SID (21 patients; 105 samples). As a result, the estimated sample size of 130 would be sufficient to observe the significant correlation between the measured pH and the strong ion difference.

Data were presented as frequency (percentage), mean ± SD, median [IQR] as appropriate. Pearson correlation (R) and the coefficient of determination (R^2^) between the measured pH and the physicochemical parameters were obtained by SPSS statistics 17.0. p-value < 0.05 was considered statistically significant.

### Results

A total of 1338 paired measures were obtained from 130 pediatric patients who admitted to PICU of Ramathibodi Hospital during 2014–2016. After exclusion of acid–base disturbances from respiratory causes, a total of 743 paired measures was subjected to the metabolic subgroup analysis. The demographic data was illustrated in Table 1. Of these patients, the most common causes of PICU admission were severe sepsis or septic shock (17.7%) and postoperative care (29.2%). Approximately 50% of patients had one or more organ failures at the time of PICU admission.

**Table 1:** Demographic data of patients whose laboratory results were included in the study

Patient characteristics	Total n = 130 patients
Male gender, n (%)	61 (47)
Age (year), median [IQR]	5.0 [2, 10]
The cause of PICU admission, n (%)	
Severe sepsis or septic shock	23 (17.7)
Hypovolemic or hemorrhagic shock	4 (3.1)
Anaphylaxis or distributive shock	1 (0.8)
Heart failure or cardiogenic shock	8 (6.2)
Post-cardiac arrest	4 (3.1)
Respiratory failure	18 (13.8)
Liver failure or hepatic encephalopathy	3 (2.3)
Upper gastrointestinal bleeding	5 (3.9)
Renal failure	3 (2.3)
Diabetes ketoacidosis	2 (1.5)
Organic acidemia	1 (0.8)
Status epilepticus	8 (6.2)
Post-operative	38 (29.1)
Others	12 (9.2)
Numbers of paired specimen per patient, median [IQR]	6 [2, 14]

**Fig. 1:**
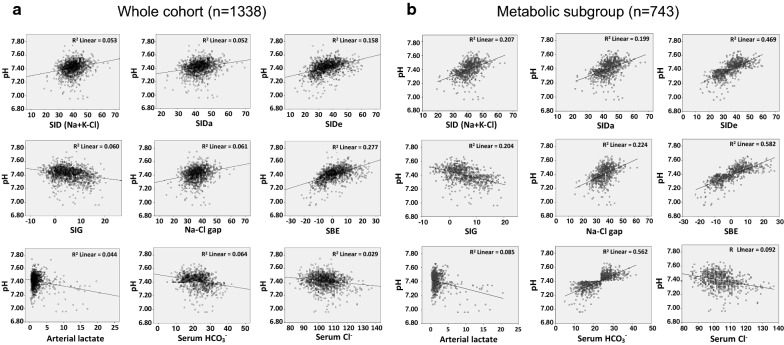
Linear correlation between the measured pH and various acid–base parameters. **a** the whole cohort analysis (n = 1338 samples). **b** the metabolic subgroup analysis (n = 743 samples). The coefficient of determination (R^2^) was shown for each pair

Among acid–base parameters that based on the physicochemical properties, SIDe showed the greatest correlation with the measured pH in the whole cohort (R^2^ = 0.158; Fig. 1a) and the metabolic subgroup analyses (R^2^ = 0.469; Fig. 1b). The simplified SID, SIDa, SIG, and Na–Cl gap also had positive, even though weaker, correlation with the measured pH (Fig. 1a, b). The traditional acid–base parameter that demonstrated the best correlation with the measured pH was SBE with R^2^ = 0.277 from the whole cohort and R^2^ = 0.582 from the metabolic subgroup (Fig. 1a, b). Interestingly, serum HCO_3_^−^ which has been routinely used as a convenient screening of acid–base disturbances, showed a small degree of correlation with the measured pH in the whole cohort analysis (R^2^ = 0.171), but not the metabolic subgroup (R^2^ = 0.562). Note that there was absence to low correlation between the measured pH and serum chloride or the arterial lactate in this study (Fig. 1a, b). Pearson correlation (R) and significant assessment of all acid–base parameters were summarized in Table 2, in which the data corresponded with those of Fig. 1a, b.

**Table 2:** Pearson correlation between the measured pH and various acid–base parameters

	Whole cohort (n = 1338 samples) R (sig 2-tailed)	Metabolic subgroup (n = 743 samples) R (sig 2-tailed)
SID	0.230 (< 0.001)	0.455 (< 0.001)
SIDa	0.227 (< 0.001)	0.446 (< 0.001)
SIDe	0.398 (< 0.001)	0.685 (< 0.001)
SIG	− 0.245 (< 0.001)	− 0.452 (< 0.001)
Na–Cl gap	0.247 (< 0.001)	0.473 (< 0.001)
SBE	0.526 (< 0.001)	0.763 (< 0.001)
Arterial lactate	− 0.210 (< 0.001)	− 0.292 (< 0.001)
Serum HCO_3_^−^	0.413 (< 0.001)	0.749 (< 0.001)
Serum Cl^−^	− 0.170 (< 0.001)	− 0.303 (< 0.001)

### Discussion

Since the physicochemical approach to acid–base status was introduced by Peter Stewart in 1981, it had been subjected to several studies, update, and refinement [7, 8, 18, 19]. However, most of the studies and clinical applications using the physicochemical approach were restricted to adult subjects [1, 2, 9–13]. There were studies showing promises of physicochemical approach in pediatric population [14, 15, 20–22]; however, those studies focused only on the association between physicochemical parameters and the clinical endpoints, but not the correlation with the measured pH directly. This study, therefore, investigated the correlation between the measured pH from blood gas analysis and several physicochemical parameters in critically ill pediatric population.

The degree of correlation varied among parameters, with SBE showing the greatest correlation with the measured pH while the single parameters such that of serum chloride and arterial lactate revealed the least association. This might reflect that single parameter alone might not be adequate in term of determination of complex acid and base disturbances in PICU. The SIDe showed the best correlation regarding physicochemical approach; nevertheless, this parameter involved with a complicated equation. In a practical standpoint, the simplified SID and Na–Cl gap which showed moderate correlation with the measured pH, especially in the metabolic subgroup, might be applicable as the convenient screening tools of acid–base derangement.

Our findings supported a possibility that Stewart’s physicochemical approach is applicable as a tool to preliminarily evaluate acid–base status in pediatric patients, mainly where arterial puncture and blood gas analysis are not commonly performed. The correlation between the measured pH and the physicochemical parameters were stronger than that of serum bicarbonate, particularly in the metabolic subgroup. Nonetheless, the SIDe and SIG still need the measured pH in their formula, and the calculation is very complicated. Only the simplified SID, SIDa, and the Na–Cl gap should be considered as the sole physicochemical approach, which is convenient at the bedside applications. Of these, the Na–Cl gap exhibited a higher correlation with the measured pH than the simplified SID and SIDa. The applicability and feasibility of the Na–Cl gap in replacement of serum bicarbonate as the screening tool of acid–base disturbances in pediatric patients should also be evaluated in the future.

In conclusion, this study provided direct evidence of the positive correlation between physicochemical parameter and blood pH in pediatric subjects. We hope this finding will encourage more study utilizing the physicochemical approach of acid–base status in pediatric patients.

## Limitations

No clinical correlation (i.e., the length of PICU and hospital stay, morbidity or mortality) with the acid–base determination using physicochemical approach was observed in this study.

## Supplementary information

**Additional file 1: Table S1.** Raw data of the measured pH from blood gas analysis and physicochemical parameters in the whole cohort (n = 1338 samples).

**Additional file 2: Table S2.** Raw data of the measured pH from blood gas analysis and physicochemical parameters in the metabolic subgroup (n = 743 samples).

## Data Availability

The datasets containing the blood gas pH and physicochemical parameters from the whole cohort (n = 1338) and the metabolic subgroup (n = 743) that support the findings of this study are made available as Additional files 1, 2.

## Abbreviations

PICU: pediatric intensive care unit
SID: strong ion difference
SIDa: apparent strong ion difference
SIDe: effective strong ion difference
SBE: standard base excess
